# Stable and Efficient Red Perovskite Light-Emitting Diodes Based on Ca^2+^-Doped CsPbI_3_ Nanocrystals

**DOI:** 10.34133/2021/9829374

**Published:** 2021-12-06

**Authors:** Wei Shen, Jianbin Zhang, Ruimin Dong, Yanfeng Chen, Liu Yang, Shuo Chen, Zhan Su, Yujun Dai, Kun Cao, Lihui Liu, Shufen Chen, Wei Huang

**Affiliations:** ^1^State Key Laboratory of Organic Electronics and Information Displays & Institute of Advanced Materials (IAM), Nanjing University of Posts & Telecommunications, 9 Wenyuan Road, Nanjing 210023, China; ^2^Frontiers Science Center for Flexible Electronics (FSCFE), MIIT Key Laboratory of Flexible Electronics (KLoFE), Northwestern Polytechnical University, Xi'an 710072, China

## Abstract

*α*-CsPbI_3_ nanocrystals (NCs) with poor stability prevent their wide applications in optoelectronic fields. Ca^2+^ (1.00 Å) as a new B-site doping ion can successfully boost CsPbI_3_ NC performance with both improved phase stability and optoelectronic properties. With a Ca^2+^/Pb^2+^ ratio of 0.40%, both phase and photoluminescence (PL) stability could be greatly enhanced. Facilitated by increased tolerance factor, the cubic phase of its solid film could be maintained after 58 days in ambient condition or 4 h accelerated aging process at 120°C. The PL stability of its solution could be preserved to 83% after 147 days in ambient condition. Even using UV light to accelerate aging, the T_50_ of PL could boost 1.8-folds as compared to CsPbI_3_ NCs. Because Ca^2+^ doping can dramatically decrease defect densities of films and reduce hole injection barriers, the red light-emitting diodes (LEDs) exhibited about triple enhancement for maximum the external quantum efficiency (EQE) up to 7.8% and 2.2 times enhancement for half-lifetime of LED up to 85 min. We believe it is promising to further explore high-quality CsPbI_3_ NC LEDs via a Ca^2+^-doping strategy.

## 1. Introduction

All-inorganic perovskite (CsPbX_3_, X = Cl, Br, I) nanocrystals (NCs) have the potential to promote the development of the luminescence and display industry due to their high photoluminescence quantum yields (PLQYs), high color purity, and solution processability [1–11]. Currently, green, red, and near-infrared perovskite light-emitting diodes (LEDs) have met the need of commercial demands from the efficiency factor (EQE > 20%) [2, 12], whereas CsPbX_3_ NC-based LEDs are still underdeveloped. It should be noted that the high-performance optoelectronic properties of CsPbX_3_ NCs are dominated by their crystal phase, especially for CsPbI_3_ [13–17]. Generally, cubic (*α*) CsPbI_3_ (direct band gap, E_g_ = 1.73 eV) exhibits good optoelectronic performance. However, the tolerance factor (*τ*) of *α*-CsPbI_3_ is too small to stabilize its cubic phase at room temperature. The metastable state of *α*-CsPbI_3_ can be easily transformed into the orthorhombic (*δ*) phase (E_g_ = 2.82 eV) with poor optoelectronic performance [3, 18–21]. Therefore, improving phase stability of *α*-CsPbI_3_ is the foundation for achieving high-performance CsPbI_3_ NC LEDs [22].

According to the Goldschmidt tolerance factor (*τ*) function for perovskite (ABX_3_), doping ions with suitable size can realize precise tuning of *τ* to stabilize cubic phase [23, 24]. Based on this principle, A-, B-, or X-site doping can maintain *α*-CsPbI_3_. It should be noted that common methods to synthesize CsPbI_3_ NCs need high temperature (>170°C) [6]. As a result, organic A-site ions (methylamine or formamidine) will be easily decomposed at high temperature [24, 25]. Currently, B- or X-site doping is the hotspot for stabilization of *α*-CsPbI_3_. Due to the ionic nature of CsPbI_3_, X-site ions are at the corner of the PbI_6_^4−^ octahedron, which can easily migrate. With Br^−^ doping as an example, though CsPbBr_x_I_3-x_ NCs exhibit a cubic phase, the ion migration of Br^−^ and I^−^ easily leads to phase separation and degradation of optoelectronic performance [26], whereas B-site ions are at the center of the PbI_6_^4−^ octahedron, which can hardly migrate. Therefore, B-site doping is the efficient way to stabilize *α*-CsPbI_3_ without any side effects, such as thermal decomposition, or phase separation [27–30]. On the basis of the Goldschmidt tolerance factor function, doping with small-sized B-site ions can efficiently stabilize *α*-CsPbI_3_ (Pb^2+^ radius 1.19 Å). For example, Mn^2+^ (0.67 Å) doping by direct synthesis or posttreatment methods can improve the stability of *α*-CsPbI_3_ NCs from a few days to nearly a month under ambient conditions [31, 32]. In addition, B-site doping can not only enhance the stability of CsPbI_3_ NCs but also boost their optoelectronic performance. Several B-site doping ions have been studied to promote the development of CsPbI_3_ NC LEDs, such as Mn^2+^ [33], Zn^2+^ [34, 35], Zr^2+^ [36], Y^3+^ [35], Cu^2+^ [37], Ni^2+^ [38], and Sr^2+^ [39–41]. It should be emphasized that some doping ions, such as Zn^2+^, Cu^2+^, and Sr^2+^, can reduce charge injection barriers and enhance carrier transport properties. To date, the state of the art for CsPbI_3_ NC LEDs is facilitated by alkaline earth metal ion doping such as Sr^2+^ doping [39–41], which has the potential to satisfy the commercial demands [23]. It should be noted that the size of Sr^2+^ (1.18 Å) is almost the same as Pb^2+^ (1.19 Å). In other words, both stability and optoelectronic performance have more room to improve by doping other small size alkaline earth metal ions, such as Ca^2+^ (1.00 Å).

Herein, we explored Ca^2+^ as a new B-site doping ion to boost CsPbI_3_ NC performance with both improved phase stability and optoelectronic properties. Doping Ca^2+^ to partly replace Pb^2+^ must increase *τ*. Therefore, Ca^2+^ doping can dramatically improve the phase stability of CsPbI_3_ NCs, which suppresses the decreasing PLQY resulting from phase transition [33, 34, 40, 42]. Systematical studies on stability were done by tuning Ca^2+^/Pb^2+^ ratios (0%, 0.35%, 0.40%, and 1.20%). For the case of 0.40% Ca^2+^/Pb^2+^ ratios, Ca^2+^-doped CsPbI_3_ NCs showed enhanced phase stability to long-term storage and heat. Its solid film could exhibit the cubic phase after 58-day storage in ambient condition or 4 h accelerated aging process at 120°C. Additionally, its PL intensity in solution decayed less than 17% after 147-day storage in ambient condition. Even using UV light to accelerate aging, the half-lifetime of PL could boost 1.8-folds as compared to that of CsPbI_3_ NCs. Furthermore, the Ca^2+^-doped CsPbI_3_ NCs were employed as the emission layer to fabricate LED. Benefitting from the Ca^2+^ doping, the defect densities were decreased to 21%, its valence band maximum went up to -5.55 eV to reduce hole injection barriers, and its lower Fermi level enhanced hole transport efficiency. As a result, Ca^2+^-doped CsPbI_3_ NC-based LED exhibited about triple enhancement for maximum EQE up to 7.8%, and 2.2 times enhancement for half-lifetime of LED up to 85 min. We believe it is promising to further explore high-quality CsPbI_3_ NC LED via Ca^2+^-doping strategy.

## 2. Result and Discussion

**Figure 1:**
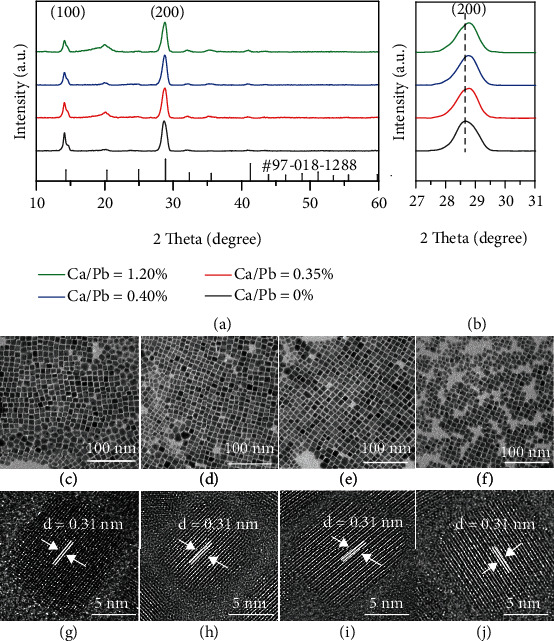
(a) XRD patterns of Ca^2+^-doped CsPbI_3_ NCs, (b) fine XRD patterns in the region of 27-31°, (c–f) TEM images of Ca^2+^-doped CsPbI_3_ NCs, (g–j) HRTEM images of Ca^2+^-doped CsPbI_3_ NCs (Ca/Pb = 0%, 0.35%, 0.40%, 1.20%).

Ca^2+^-doped CsPbI_3_ NCs were synthesized by using different feed ratios of Ca(Ac)_2_/PbI_2_ (0%, 15%, 25%, and 35%) via hot injection method (Experimental Section). As shown in Table S1, the real Ca^2+^/Pb^2+^ ratios for Ca^2+^-doped CsPbI_3_ NCs were determined by ICP-MS. According to ICP-MS results, the real Ca^2+^/Pb^2+^ ratios were 0%, 0.35%, 0.40%, and 1.20% for the condition of 0%, 15%, 25%, and 35% feed ratios of Ca(Ac)_2_/PbI_2_. Such results implied that Ca^2+^ was successfully doped into CsPbI_3_ NCs. The Pb^2+^ is located in the center of the octahedron (PbI_6_^4-^). The exchange and migration of the B-site ion is more difficult as compared to that of A-site and X-site ions. Therefore, B-site ions do not easily incorporate into the CsPbI_3_ lattice, and the actual doping ratio of the B-site ion is much lower than the feed ratio [18, 34, 38, 40, 43]. The size of Ca^2+^ (1.00 Å) is smaller than Pb^2+^ (1.19 Å), and Ca^2+^ doping must induce the lattice contraction, which can be confirmed by XRD.  Figure 1(a) shows XRD patterns for Ca^2+^-doped CsPbI_3_ NC films with different real Ca^2+^/Pb^2+^ ratios. The patterns for all films match well with *α*-CsPbI_3_ (PDF#97-018-1288), and the diffraction peaks at 14.09° and 28.58° correspond to the (100) and (200) planes, respectively [34]. It should be emphasized that the splitting peaks at around 14° may be related to *γ*-CsPbI_3_ [44, 45], while the characterization patterns for *γ*-CsPbI_3_ from 25-30° cannot be observed. It is possible that a slight lattice distortion occurred due to the influence of water and oxygen during the test [20, 46]. With increasing Ca^2+^/Pb^2+^ ratio, no additional diffraction peaks were observed, which indicated that doping Ca^2+^ did not significantly change the CsPbI_3_ crystal phase. Their fine XRD patterns are shown in Figure 1(b). There is a slight shift toward a higher diffraction angle for the *α*-CsPbI_3_ (200) plane (28.62° for 0%, 28.77° for 0.35%, 28.78° for 0.40%, and 28.82° for 1.20%), which verifies the lattice contraction resulted from the partial replacement of Pb^2+^ (1.19 Å). An enhanced peak at 20° is attributed to the (110) crystal plane for CsPbI_3_. Based on the HRTEM images, the cubic NCs become truncated cubic NCs (Figure S1a-d). Therefore, (110) crystal planes are more exposed and enhance the peak at 20° [47, 48]. Furthermore, the morphology and size characterization for Ca^2+^-doped CsPbI_3_ NCs were studied by TEM. As shown in Figures 1(c)–1(f), with increasing Ca^2+^/Pb^2+^ ratio, Ca^2+^-doped CsPbI_3_ NCs maintain a cubic shape with uniform size distribution. The average size decreased from 11.11 ± 0.90 nm, 10.62 ± 0.98 nm, 10.40 ± 1.00 nm, and 9.89 ± 1.07 nm (Figure S2), which is mainly because Ca^2+^ doping influences lattice contraction or nucleation and growth processes. HRTEM images (Figures 1(g)–1(j)) reveal that Ca^2+^-doped CsPbI_3_ NCs are highly crystalline with displayed lattice fringes. The lattice distances of all samples are 0.31 nm corresponding to the (200) plane of *α*-CsPbI_3_ [49]. This is due to the limited TEM accuracy, which makes it difficult to distinguish the difference of 0.001 nm. According to the XRD test results and Scherer's formula, the lattice distances of (200) are 0.311 nm (0%), 0.310 nm (0.8%), 0.310 nm (3.1%), and 0.309 nm (5.0%). Therefore, only XRD results can confirm that Ca^2+^ is successfully doped into CsPbI_3_ NCs.

**Figure 2:**
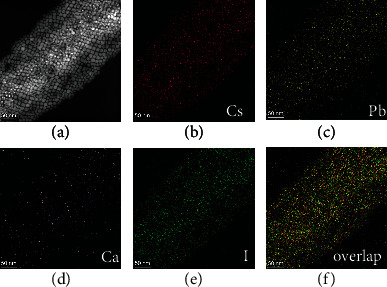
(a) HAADF-STEM images of Ca^2+^-doped CsPbI_3_ NCs and the corresponding elemental mapping images of Cs (b), Pb (c), Ca (d), I (e), and merged image (f). (b–f) Ca/Pb = 0.40%.

Furthermore, the elemental mapping images were measured by EDS (Figure S3a-d). We chose the elemental mapping area of Ca^2+^-doped CsPbI_3_ NCs in a high-angle annular dark field scanning transmission electron microscopy image (HAADF-STEM) (Figure 2(a)). Elemental mapping images of Cs, Pb, Ca, and I (Figures 2(b)–2(e)) can be observed clearly. We merged these elemental mapping images to obtain the overlapped image (Figure 2(f)), which shows that the positions of Cs, Pb, Ca, and I are uniformly distributed in NCs. Therefore, these results can directly demonstrate that Ca^2+^ is doped in CsPbI_3_ NCs.

**Figure 3:**
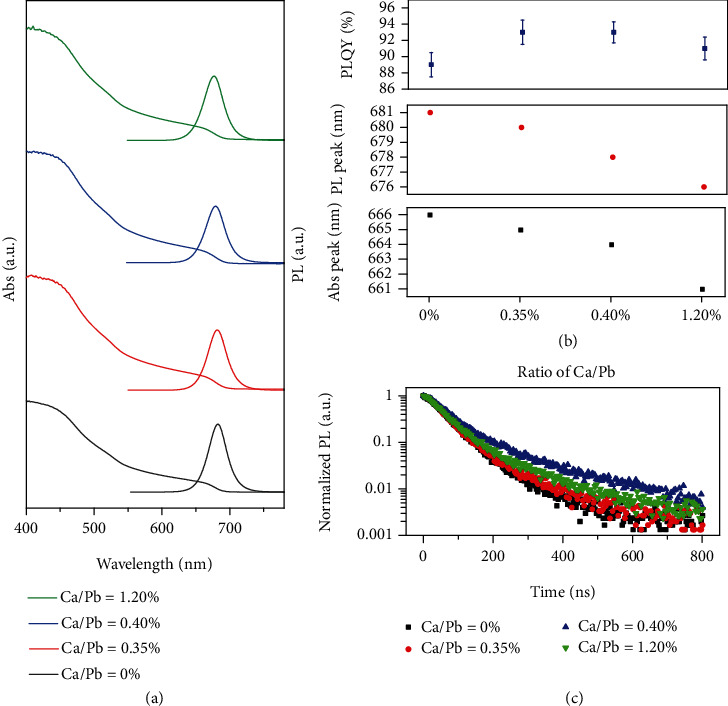
UV-vis absorption and PL spectra (a); UV-vis absorption peaks, PL peaks, and PLQYs (b); PL decay curves (c) of Ca^2+^-doped CsPbI_3_ NC solutions with different Ca/Pb ratios (Ca/Pb = 0%, 0.35%, 0.40%, 1.20%).

The optical properties of Ca^2+^-doped CsPbI_3_ NCs are shown in Figure 3. The UV-vis absorbance and PL spectra of Ca^2+^-doped CsPbI_3_ NCs are compared in Figure 3(a) and Figure S4a-b. With the ratio of Ca/Pb increasing, both of their UV-vis absorption and PL peaks appear as blue shifts due to their lattice contraction, size decrease, and Ca^2+^ orbitals to influence the electronic structure of NCs [34, 40, 50, 51] (Figure S4a-b and Figure S2). Figure 3(b) summarizes the UV-vis absorption peaks, PL peaks, and PLQY evolution with different ratios of Ca/Pb. The UV-vis absorption peaks exhibit a blue shift from 666 nm to 665, 664, and 661 nm with the ratio of Ca/Pb increasing from 0% to 0.35%, 0.40%, and 1.20%, respectively. Simultaneously, their PL peaks also exhibit a blue shift from 682 nm to 676 nm with increase of the ratio of Ca/Pb. Furthermore, benefitting from Ca^2+^ doping, their PLQYs can be improved. With increasing Ca/Pb ratios from 0% to 0.40%, their PLQYs increased from 89% (±1.5%) to 93% (±1.3%), whereas further increasing the Ca/Pb ratio to 1.20% led PLQY lower to 91% (±1.4%). This is due to that the smaller NCs (Ca/Pb = 1.20%) with larger surface-to-volume ratio must have more surface defects. In addition, Ca^2+^ cannot be as emission centers. Therefore, excess Ca^2+^ doping may decrease PLQY. Figure 2(c) shows the decay curves of Ca^2+^-doped CsPbI_3_ NCs. All of the decay curves can be fitted by a double-exponential (Table S2). The average PL lifetimes of Ca^2+^-doped CsPbI_3_ NCs are 71.76 ns (0%), 77.47 ns (0.35%), 119.47 ns (0.40%), and 92.62 ns (1.20%). Such results demonstrate that the improved *τ* facilitated by Ca^2+^ doping can reduce the lattice distortion and phase transition to maintain PLQYs at a relatively high level.

**Figure 4:**
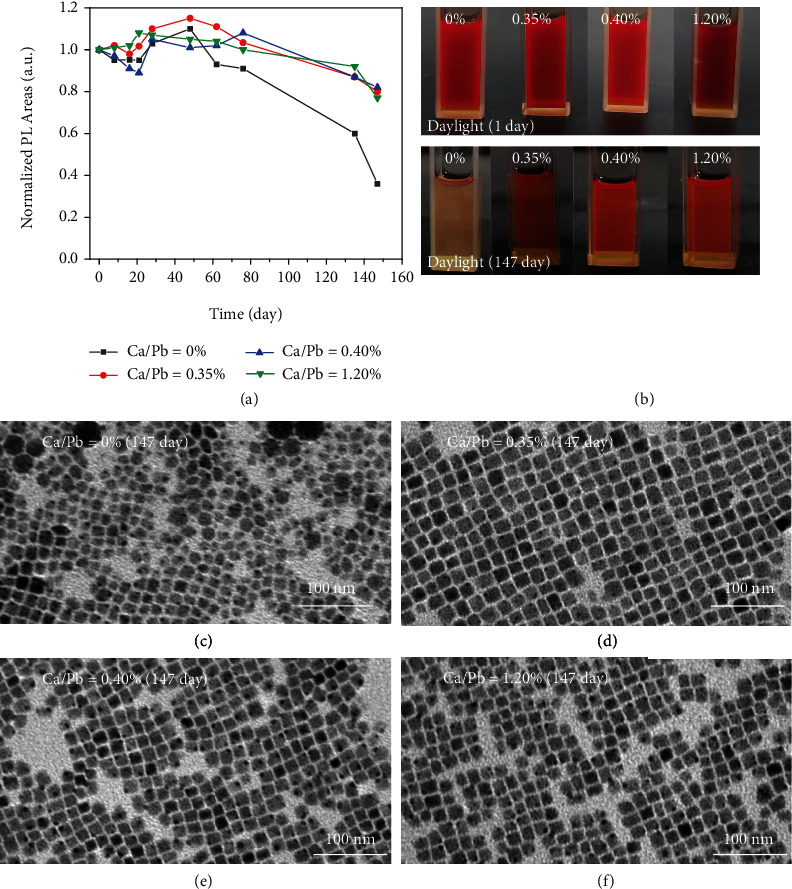
(a) The PL area evolution with increasing storing time in ambient condition; (b) images of the Ca^2+^-doped CsPbI_3_ NC solutions under daylight before and after 147-day storage (from left to right: Ca/Pb = 0%, 0.35%, 0.40%, 1.20%), and (c–f) TEM images of Ca^2+^-doped CsPbI_3_ NCs after 147-day storage.

In addition to boosting their PLQYs, the increase in *τ* for Ca^2+^ doping in CsPbI_3_ can much improve their stability. We measured the stability of their solutions and solid films. Firstly, all samples were stored in the conditions of 20 ± 5°C and 40−50% humidity.  Figure 4(a) shows PL intensity evolution with storage time. After a 147-day storage, PL intensity of CsPbI_3_ NCs decreased to 36% of the initial one, whereas PL stabilities of Ca^2+^-doped CsPbI_3_ NCs exhibited much improvement. After 147-day storage, an obvious color bleaching was observed for the CsPbI_3_ NC solution, while the red color of Ca^2+^-doped CsPbI_3_ NC solutions can be maintained (Figure 4(b)). PL intensities of Ca^2+^-doped CsPbI_3_ NC solutions can be preserved to 80% (0.35%), 83% (0.40%), and 76% (1.20%) of the initial intensities after 147-day storage (Figure 4(a) and Figure S5). We further studied their morphology changes by TEM. The size of CsPbI_3_ NCs became nonuniform and noncubic shape and tended to be aggregated, which must lead to their PL decrease (Figure 4(c) and Figure S5). In contrast, cubic shape and uniform size distribution of Ca^2+^-doped CsPbI_3_ NCs can be kept with relatively high PL performance (Figure 4(c) and Figure S5). Secondly, Ca^2+^-doped CsPbI_3_ NCs exhibited improved stability against to UV. All of the samples were placed under 365 nm UV light (8 W), and their PL intensities decayed with UV irradiation time increasing. We periodically measured their PL spectra, which were used to calculate their half-lifetimes (T_50_) under UV light (Figure S6 and Figure S7a). On the basis of these data, T_50_ were 52 min (0%), 74 min (0.35%), 92 min (0.40%), and 85 min (1.20%), separately. About 1.8 times enhancement of UV stability was observed for the 0.40% Ca/Pb ratio. Further increasing UV irradiation time to 100 min, it was hard to observe the red color for CsPbI_3_ NCs, while Ca^2+^-doped CsPbI_3_ NCs could still emit red PL (Figure S7b). As a result, Ca^2+^-doped CsPbI_3_ NCs exhibit enhanced UV stability as compared to CsPbI_3_ NCs.

**Figure 5:**
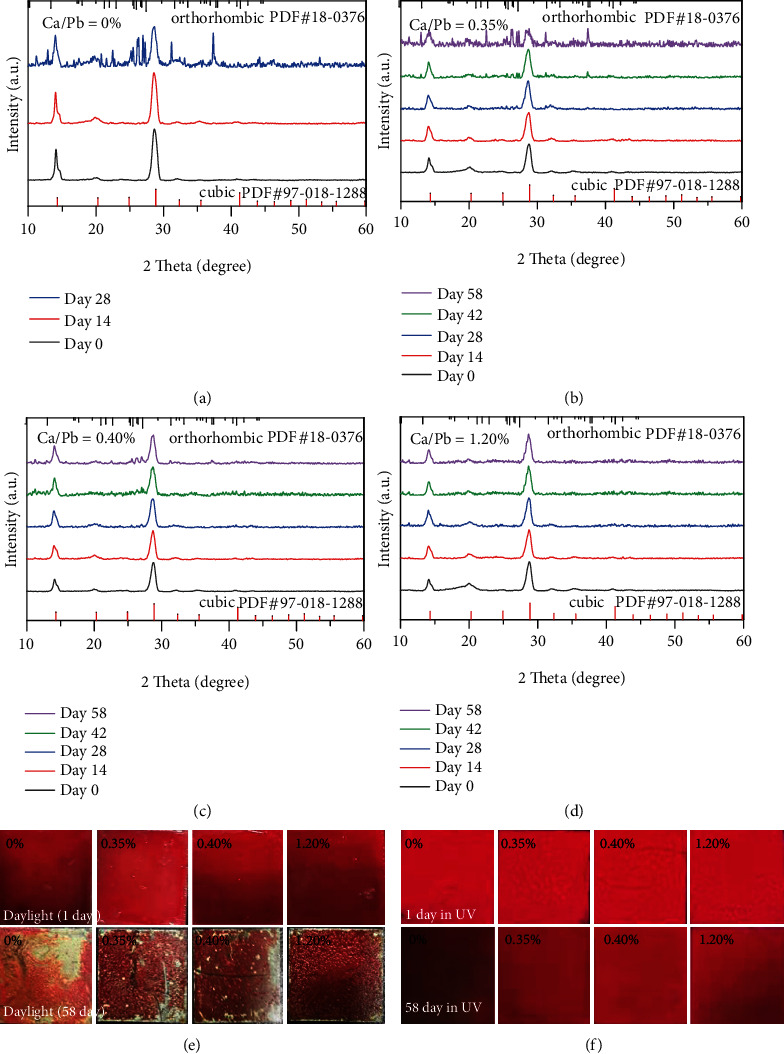
(a–d) XRD pattern evolution for Ca^2+^-doped CsPbI_3_ films in ambient condition (40−50% RH at 20 ± 5°C); images of Ca^2+^-doped CsPbI_3_ films under daylight (e) and under UV light (f) before and after 58-day storage in ambient condition.

According to the above results, Ca^2+^-doped CsPbI_3_ NC solutions exhibited improved stability in ambient condition and UV light, whereas the application of these NCs must form solid films. Therefore, we further studied their stability in solid films, such as in ambient condition (40−50% RH at 20 ± 5°C) and heating condition (120°C). As shown in Figure 5(a), *α*-CsPbI_3_ (cubic phase) film gradually converted to *δ*-CsPbI_3_ (orthorhombic phase) film after 28-day storage time, and its color changed from red to yellow (Figure 5(e)). However, Ca^2+^-doped CsPbI_3_ films exhibited better crystal phase stability. The crystal phase of their films (Ca/Pb = 0.40%, 1.20%) could be maintained to the cubic phase after 58-day storage, which exhibited good PL performance (Figures 5(e) and 5(f)). Such results demonstrate that the phase stability of CsPbI_3_ can be enhanced in ambient condition facilitated by Ca^2+^ doping. Additionally, we verified their thermal stability in the solid state. All the films were placed on a hot plate at 120°C, and we periodically measured their XRD patterns. The CsPbI_3_ film exhibited the poorest phase stability, and its phase gradually converted to the orthorhombic phase after 120°C heating for 2 h (Figure S8). On the basis of previous reports, the small size effect can reduce CsPbI_3_ lattice distortion to partly prevent phase transition [39, 52]. As an ionic nature of CsPbI_3_, all ion migrations can be accelerated at high temperature. As a result, ion migration and crystal fusion easily occur in the solid state, which leads to increasing the size of CsPbI_3_ NCs. The enlarged size of CsPbI_3_ must induce phase transition [14, 53, 54]. Benefitting from small-sized Ca^2+^ doping, the increase in *τ* should improve the phase stability of CsPbI_3_. With increase of the Ca/Pb ratio from 0% to 1.20%, their cubic phase could be maintained at least for 4 h at 120°C (Figure S8). Therefore, the thermal stability of CsPbI_3_ films can be enhanced via Ca^2+^ doping.

**Figure 6:**
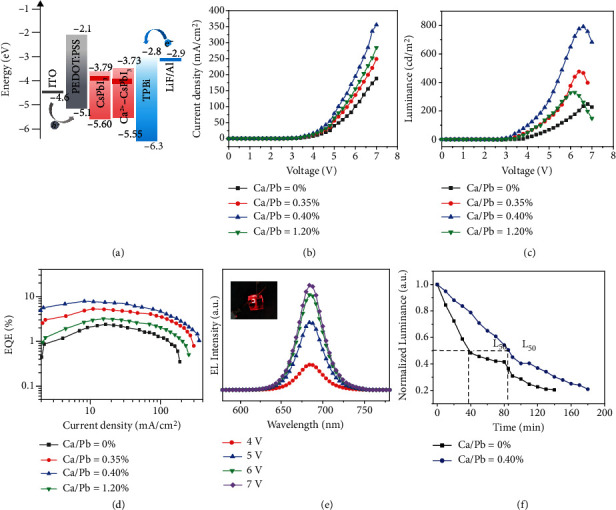
(a) LED energy-level diagram, (b) current density, (c) luminescence, and (d) EQEs for LEDs; (e) EL spectra of Ca^2+^-doped CsPbI_3_ NCs (0.40%) LEDs on various driving voltages and a photograph of the working device (inset in (e)); (f) operational stability of the LEDs at a constant current density of 5.0 mA/cm^2^.

To identify the positive effect of Ca^2+^-doped CsPbI_3_ NCs for LEDs, we used Ca^2+^-doped CsPbI_3_ NC films as emitting layers to fabricate red LEDs.  Figure 6(a) is the energy-level diagram of LED. The LED architecture consists of a multiple-layered structure of ITO (170 nm)/PEDOT:PSS (30 nm)/Ca^2+^-doped CsPbI_3_ NCs (30 nm)/TPBi (40 nm)/LiF (1 nm)/Al (100 nm) shown in Figure S9. Herein, ITO is the anode; PEDOT:PSS is the hole-injection layer; TPBi and LiF are the electron-transport and electron-injection layer, respectively; and Al is the cathode.  Figure 6(b) presents the current density as functions of voltage. The LED with Ca^2+^-doped CsPbI_3_ NCs (Ca/Pb = 0.40%) exhibited obviously enhanced current density, which implied the decreasing of defect density as well as the improvement for carrier transport efficiency.

Firstly, hole-only devices with a structure of ITO/PEDOT:PSS/CsPbI_3_ NCs or Ca^2+^-doped CsPbI_3_ NCs/MoO_3_/Al (Figure S10) were used to quantitatively measure the defect density of NCs films. The defect density (*N*_*t*_) is calculated according to the following equation:

(1) $$\begin{array}{c} N_{t}=\frac{2\varepsilon \varepsilon_{0}V_{\text{TFL}}}{eL^{2}}, \end{array}$$

where *ε*_0_ and *ε* are the vacuum dielectric constant and the relative dielectric constant, respectively; *V*_TFL_ is the trap-filled limit voltage; *L* is the thickness of the NC film; and *e* is the elementary electronic charge. By assuming that *ε* = 6.3 [34], the defect densities of CsPbI_3_ and Ca^2+^-doped CsPbI_3_ NC films were 0.71 × 10^17^ and 0.15 × 10^17^ cm^−3^, respectively. This means that the defect density could be decreased to 21.1% facilitated by 0.40% Ca^2+^ doping. Such results match with those results of PLQY and TRPL. In addition, the hole migration rates were estimated by fitting the space-charge-limited-current region (SCLC) with Child's law:

(2) $$\begin{array}{c} J=\frac{9\varepsilon \varepsilon_{0}\mu V^{2}}{8L^{3}}, \end{array}$$

where *ε*_0_ is the vacuum permittivity; *ε*_*r*_ is the average relative dielectric constant of CsPbI_3_ (*ε*_*r*_ ≈ 6.32); *L* is the thickness of the perovskite film; and *J*, *μ*, and *V* are the measured current density, carrier migration rates, and applied voltage, respectively [34]. The hole mobilities of CsPbI_3_ and Ca^2+^-doped CsPbI_3_ NC films were 2.8 × 10^−8^ and 6.0 × 10^−8^ cm^2^V^−1^ s^−1^, respectively. With the Ca^2+^ doping, the hole mobility increased, which enhanced hole transport efficiency.

Then, the improvement for carrier transport efficiency can be confirmed by ultraviolet photoelectron spectra (UPS). Combining with their optical bandgaps (E_g_) for Ca^2+^-doped CsPbI_3_ NC film (Figure S11a) and their UPS spectra of CsPbI_3_ NCs (Figure S11b), their conduction band minimum (CBM), valence band maximum (VBM), and Fermi level can be obtained. For CsPbI_3_ NCs, its CBM, VBM, and Fermi level were -3.79 eV, -5.60 eV, and -3.93 eV, respectively. Benefitting from Ca^2+^ doping (0.40%), its CBM, VBM, and Fermi level shifted to -3.73 eV, -5.55 eV, and -4.05 eV, respectively. The higher VBM reduces the energy barrier between PEDOT:PSS and the emission layer to boost hole transport efficiency. Additionally, the lower Fermi level reveals the transformation of CsPbI_3_ NCs from n-type to a more nearly ambipolar nature facilitated by Ca^2+^ doping, which also enhances hole transport efficiency [34, 37].

Benefitting from the enhanced current density, the Ca^2+^-doped CsPbI_3_ NC LEDs exhibited stronger brightness than CsPbI_3_ NC LEDs in the whole driving voltage range (Figure 6(c) and Figure S12). In the case of 0.40% Ca^2+^ doping, LED had a maximum luminance of 790 cd/m^2^ (7.2 V), which was doubled as compared to the nondoping one. In addition, the maximum and average EQEs for Ca^2+^-doped CsPbI_3_ NC (0.40%) LEDs were 7.8% and 6.3%, both of which enhanced to about 3 times as compared to the nondoping one, respectively (Figure 6(d) and Figure S13). Therefore, our work using Ca^2+^ as a new B-site doping ion to boost CsPbI_3_ NC LEDs is quite promising (Table S3). Their electroluminescent (EL) spectra exhibit stable and sharp peaks at 683 nm with a full width at half-maximum (FWHM) of 35 nm on various driving voltages (Figure 6(e) and Figure S14). A bright red emission could be observed at 5.0 V (inset in Figure 6(e)), and its Commission Internationale del'Eclairage (CIE) color coordinate was (0.72, 0.27) (Figure S15). Furthermore, the operational stability of the LED was evaluated at a constant current density of 5.0 mA/cm^2^. The luminance of CsPbI_3_ NC LEDs decreases to half at 39 min, while the half-lifetime of Ca^2+^-doped CsPbI_3_ NC (0.40%) LEDs could significantly increase to 85 min. A 2.2-fold improvement for the half-lifetime of LED confirmed that Ca^2+^ doping is a powerful strategy to promote both stability of CsPbI_3_ NCs and their LEDs. In a word, the boosting of both the efficiency and the stability of LEDs is mainly attributed to a decrease in defect density, improvement on hole injection efficiency, and reduction in phase transition.

## 3. Conclusion

In summary, we explored stable and high-performance CsPbI_3_ NCs based on Ca^2+^ (1.00 Å) doping. With a Ca^2+^/Pb^2+^ ratio of 0.40%, the phase stability could be greatly enhanced. Ca^2+^-doped CsPbI_3_ NC solid films could maintain the cubic phase after 58-day storage in ambient condition or 4 h accelerated aging process at 120°C. Moreover, the PL stability could be also improved. The PL intensity of Ca^2+^-doped CsPbI_3_ NC solutions could be preserved to 83% after 147-day storage in ambient condition. Even using UV light to accelerate aging, the T_50_ of PL could be boosted 1.8-folds as compared to that of CsPbI_3_ NCs. Red LED based on Ca^2+^-doped CsPbI_3_ NCs exhibited about triple enhancement for maximum EQE up to 7.8% and 2.2 times enhancement for half-lifetime of LED up to 85 min. These were mainly attributed to the decreased defect densities of films and reduced hole injection barrier facilitated by Ca^2+^ doping. Ca^2+^ as a new B-site doping ion can efficiently boost both stability and performance for CsPbI_3_ NC LEDs, which has the potential to promote the development of CsPbI_3_ NC LEDs.

## 4. Experimental Section

### 4.1. Chemical Materials

Oleic acid (OA, 90%), 1-octadecene (ODE, 90%), methyl acetate (MeOAc, 99%), hydriodic acid (HI, 57%) oleylamine (OLA, 80-90%), cesium carbonate (Cs_2_CO_3_, 99.99%), calcium acetate (Ca(Ac)_2_, 95%), and lead iodide (PbI_2_, 99.99%) were purchased from Aladdin. Poly(3,4-ethylenedioxythiophene):poly(4-styrenesulphonate) (PEDOT:PSS, CLEVIOS P VP AI 4083) was purchased from Heraeus Materials Technology Co. Ltd. 2,2′,2^″^-(1,3,5-Benzinetriyl)-tris(1-phenyl-1-H-benzimidazole) (TPBi) was purchased from Nichem Fine Technology Co. Ltd. All the chemicals were directly used without further purification.

### 4.2. Synthesis of Cs-OA

0.39 g Cs_2_CO_3_, 18 mL ODE, and 2.0 mL OA were mixed into a 100 mL three-neck flask and dried in vacuum at 120°C for 1 h. Then, the mixture was heated to 150°C under N_2_ until the Cs_2_CO_3_ powders were completely dissolved to form a transparent solution. The solution was cooled down to room temperature via ice-water bath and preheated to 110°C before use.

### 4.3. Synthesis of OLA-HI

20 mL OLA and 2 mL HI were mixed into a 100 mL three-neck flask. Then, the solution was heated to 120°C for 2 h under vacuum to remove the water. Then, the solution was cooled down to 60°С to obtain the OLA-HI solution and preheated to 90°C before use.

### 4.4. Synthesis of Ca^2+^-Doped CsPbI_3_ NCs

In a typical synthesis, PbI_2_ (0.4 mmol), Ca(Ac)_2_ (0, 0.06, 0.10, and 0.14 mmol), and 10 mL ODE were mixed into a 100 mL three-neck flask. The mixture was degassed and dried in vacuum for 1 h at 120°C. Then, 1.0 mL OLA, OA, and preheated OLA-HI were injected into the reaction flask, separately. The mixed solution became clear and was kept under vacuum for 30 min at 120°C. Finally, the temperature was increased to 260°C, and 1.0 mL Cs-OA solution was injected immediately. After 1 min, the reaction flask was placed into an ice-water bath to stop the reaction.

### 4.5. Purification of NCs

The as-prepared NC solution was mixed with the same volume of MeOAc and was centrifuged at 15000 rpm for 5 min at 19°C to remove the supernatant. The precipitate was redispersed into toluene. Then, the NC toluene solution was mixed with the same volume of MeOAc and was centrifuged at 15000 rpm for 5 min at 19°C. After removing the supernatant, the precipitate was dispersed into toluene. Finally, the redispersed NC solution was centrifuged at 15000 rpm for 5 min at 19°C to discard the precipitate, and the supernatant was preserved for characterization and device fabrication.

### 4.6. Fabrication of LED Devices

Indium tin oxide- (ITO-) coated glass substrates were cleaned by ultrasonic cleaning for 15 min in acetone, ethanol, and deionized water, separately, and dried with nitrogen flow. The clean ITO glasses were treated by UV-ozone for 15 min. Then, PEDOT:PSS solutions were spin-coated onto the ITO substrates at 3000 rpm for 30 s and annealed at 120°C for 15 min in air. NCs were spin-coated onto the PEDOT:PSS layer at 1500 rpm for 30 s. This process was repeated five times. Finally, 40 nm of the TPBi layer, 1 nm of the LiF layer, and 100 nm of Al electrode were deposited in sequence by a thermal evaporation system under a high vacuum (∼2 × 10^−4^ Pa). The active area of the devices was 10 mm^2^ as defined by the overlapping area of the ITO and Al electrodes.

### 4.7. Characterizations

The ultraviolet-visible (UV-Vis) absorption spectra of NC solutions were measured by a PerkinElmer Lambda 35S instrument in transmission mode. PL spectra were collected by a RF6000 spectrofluorometer with an excitation wavelength of 500 nm. The PL lifetimes of NCs were measured by a FLS920 fluorescence spectrometer with a pulse laser at 375 nm. The chemical compositions were measured by a PerkinElmer NexION 2000 inductively coupled plasma mass spectrometry (ICP-MS). X-ray diffraction (XRD) data were collected by a Bruker D8 Advance X-ray powder diffractometer with Cu K*α* radiation (*λ* = 0.154 nm). Photoluminescence fluorescence quantum yield (PLQY), which is defined as the ratio of emitted photons to absorbed ones, was determined by a FLS920 fluorescence spectrometer equipped with an integrating sphere. The morphology and size of NCs were confirmed by transmission electron microscope (TEM) (Hitachi, HT7700), high-resolution TEM (HRTEM) (Talos, F200X), and energy-dispersive X-ray spectroscopy (EDS). The electroluminescent (EL) spectra and luminance- (L-) current density- (J-) voltage (V) characteristics were collected by using a Keithley 2400 source and PR-655 spectra scan spectrophotometer (Photo Research). The characterization for LED devices was measured at room temperature in air.

## Data Availability

All data needed to evaluate the conclusions in the paper are presented in the paper and/or the Supplementary Materials. Additional data related to this paper may be requested from the authors.

## Supplementary Materials

Supplementary Materials Supplementary 1. Figure S1: HRTEM images of Ca^2+^-doped CsPbI_3_ NCs. Figure S2: statistical histogram particle size. Figure S3: EDX spectrum of Ca^2+^-doped CsPbI_3_ NCs. Figure S4: normalized UV-vis absorption and normalized PL spectra. Figure S5: the PL evolution of Ca^2+^-doped CsPbI_3_ NC solutions with increase of storing time. Figure S6: the PL area evolution with increasing irradiation time. Figure S7: the PL evolution of Ca^2+^-doped CsPbI_3_ NCs under 365 nm. Figure S8: XRD patterns evolution for Ca^2+^-doped CsPbI_3_ NC thin films at 120°C. Figure S9. the cross-section SEM image of LEDs. Figure S10: J-V curves of “hole-only” devices. Figure S11: E_g_ and UPS of Ca^2+^-doped CsPbI3 NCs. Figure S12: current density and luminescence for LEDs. Figure S13: histogram of EQEs for 10 LEDs. Figure S14: (a–d) normalized EL spectra of Ca^2+^-doped CsPbI_3_ NCs. Figure S15: the corresponding CIE coordinates for the EL spectra. Table S1: ICP-MS data. Table S2: the time-resolved PL decays. Table S3: current CsPbI_3_ NC LED performance with similar structure.
