# Supplementary material for: Stable and Efficient Red Perovskite Light-Emitting Diodes Based on Ca2+-Doped CsPbI3 Nanocrystals
Source: Research (Wash D C). 2021 Dec 6;2021:9829374. doi: 10.34133/2021/9829374 (PMC8672203; doi:10.34133/2021/9829374)
Supplement: Supplementary Materials — Supplementary 1. Figure S1: HRTEM images of Ca2+-doped CsPbI3 NCs. Figure S2: statistical histogram particle size. Figure S3: EDX spectrum of Ca2+-doped CsPbI3 NCs. Figure S4: normalized UV-vis absorption and normalized PL spectra. Figure S5: the PL evolution of Ca2+-doped CsPbI3 NC solutions with increase of storing time. Figure S6: the PL area evolution with increasing irradiation time. Figure S7: the PL evolution of Ca2+-doped CsPbI3 NCs under 365 nm. Figure S8: XRD patterns evolution for Ca2+-doped CsPbI3 NC thin films at 120°C. Figure S9. the cross-section SEM image of LEDs. Figure S10: J-V curves of “hole-only” devices. Figure S11: Eg and UPS of Ca2+-doped CsPbI3 NCs. Figure S12: current density and luminescence for LEDs. Figure S13: histogram of EQEs for 10 LEDs. Figure S14: (a–d) normalized EL spectra of Ca2+-doped CsPbI3 NCs. Figure S15: the corresponding CIE coordinates for the EL spectra. Table S1: ICP-MS data. Table S2: the time-resolved PL decays. Table S3: current CsPbI3 NC LED performance with similar structure. [file 9829374.f1.docx]

**Supporting Information**

**Stable and Efficient Red Perovskite Light-Emitting Diodes based on Ca^2+^-doped CsPbI_3_ Nanocrystals**

Wei Shen ^a, 1^, Jianbin Zhang ^a, 1^, Ruimin Dong ^a^, Yanfeng Chen ^a^, Liu Yang ^a^, Shuo Chen ^a^, Zhan Su ^a^, Yujun Dai ^a^, Kun Cao ^a^, Lihui Liu ^a^, Shufen Chen ^a, *^, Wei Huang ^b *^

[a] State Key Laboratory of Organic Electronics and Information Displays & Institute of Advanced Materials (IAM), Nanjing University of Posts & Telecommunications, 9 Wenyuan Road, Nanjing 210023, China. E-mail: [iamsfchen@njupt.edu.cn](mailto:iamsfchen@njupt.edu.cn).

[b] Frontiers Science Center for Flexible Electronics (FSCFE), MIIT Key Laboratory of Flexible Electronics (KLoFE), Northwestern Polytechnical University, Xi'an 710072, China. E-mail: iamwhuang@nwpu.edu.cn.

**Contents**

**Fig. S1.** (a-d) HRTEM images of Ca^2+^-doped CsPbI_3_ NCs. (Ca/Pb = 0%, 0.35%, 0.40%, 1.20%)

**Fig. S2.** Statistical histogram particle size of Ca^2+^-doped CsPbI_3_ NCs. (a) Ca/Pb=0%, (b) Ca/Pb=0.35%, (c) Ca/Pb=0.40%, (d) Ca/Pb=1.20%.

**Fig. S3.** EDX spectrum of Ca^2+^-doped CsPbI_3_ NCs. (a) Ca/Pb=0%, (b) Ca/Pb=0.35%, (c) Ca/Pb=0.40%, (d) Ca/Pb=1.20%.

**Figure S4**. (a) Normalized UV-vis absorption (b) Normalized PL spectra of Ca^2+^-doped CsPbI_3_ NCs with different Ca/Pb ratios. (Ca/Pb = 0%, 0.35%, 0.40%, 1.20%)

**Figure S5**. The PL evolution of Ca^2+^-doped CsPbI_3_ NCs solutions with increase of storing time. (a) Ca/Pb=0%, (b) Ca/Pb=0.35%, (c) Ca/Pb=0.40%, (d) Ca/Pb=1.20%.

**Figure S6**. The PL areas evolution with increasing UV irradiation time. (a) Ca/Pb = 0%, (b) Ca/Pb = 0.35%, (c) Ca/Pb =0.40%, (d) Ca/Pb = 1.20%.

**Figure S7**. The PL evolution of Ca^2+^-doped CsPbI_3_ NCs under 365 nm (8 W) UV irradiation. (a) Ca/Pb = 0%, (b) Ca/Pb = 0.35%, (c) Ca/Pb =0.40%, (d) Ca/Pb = 1.20%.

**Figure S8**. XRD patterns evolution for Ca^2+^-doped CsPbI_3_ NCs thin films at 120 ^o^C (humidity 40%-50%). (a) Ca/Pb = 0%, (b) Ca/Pb = 0.35%, (c) Ca/Pb =0.40%, (d) Ca/Pb = 1.20%.

**Figure S9.** The cross-section SEM image of LEDs.

**Figure S10**. J-V curves of "hole-only" devices of Ca^2+^-doped CsPbI_3_ NCs. (Ca/Pb =0%, 0.40%)

**Figure S11**. (a) E_g_ of Ca^2+^-doped CsPbI_3_ NCs. (b) UPS spectra of Ca^2+^-doped CsPbI_3_ NCs. (Ca/Pb =0%, 0.40%)

**Figure S12**. (a) current density, (b) luminescence for LEDs.

**Figure S13.** Histogram of EQEs for 10 LEDs fabricated by using Ca^2+^-doped CsPbI_3_ NCs as the red-emitting layer.

**Figure S14**. EL spectra of LEDs at different voltages.

**Figure S15**. The inset shows the corresponding CIE coordinates for the EL spectra.

**Table S1**. The actual ratio of the Ca/Pb by ICP-MS for different Ca/Pb feed ratios.

**Table S2**. The time-resolved PL decays of samples with Ca^2+^-doped CsPbI_3_ NCs.

**Table S3.** Current CsPbI_3_ NCs LED performance with similar structure.


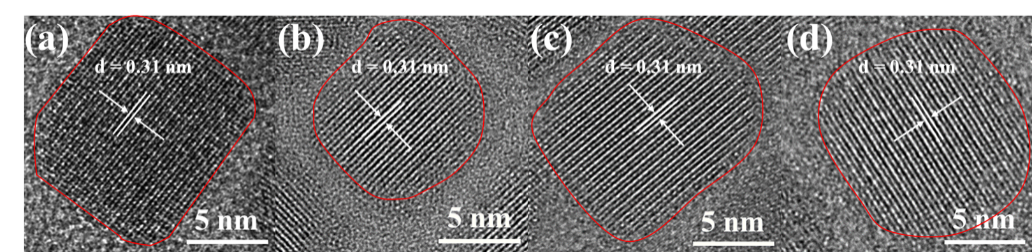


**Figure S1**. (a-d) HRTEM images of Ca^2+^-doped CsPbI_3_ NCs. (Ca/Pb = 0%, 0.35%, 0.40%, 1.20%)


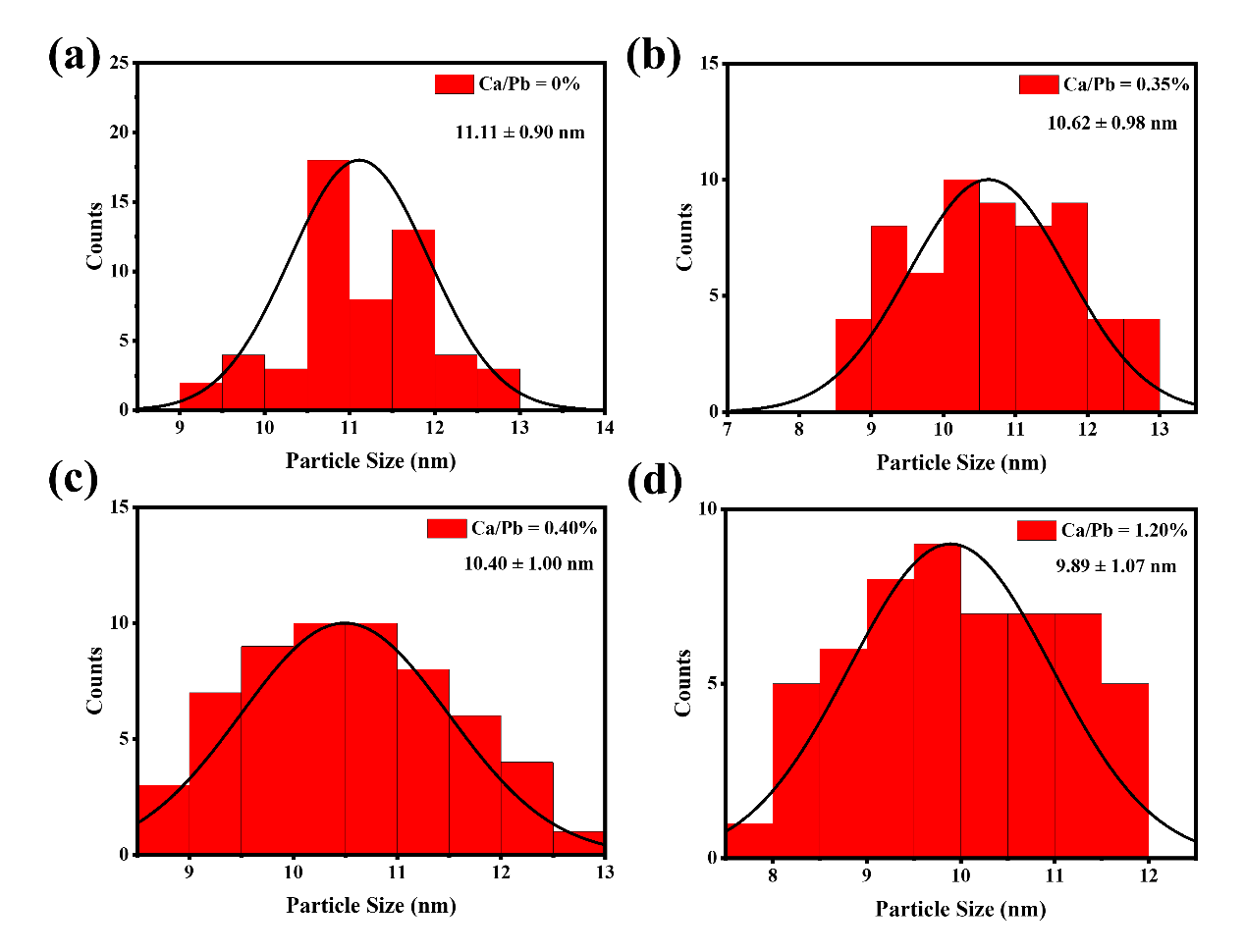


**Figure S2**. Statistical histogram particle size of Ca^2+^-doped CsPbI_3_ NCs. (a) Ca/Pb=0%, (b) Ca/Pb=0.35%, (c) Ca/Pb=0.40%, (d) Ca/Pb=1.20%.


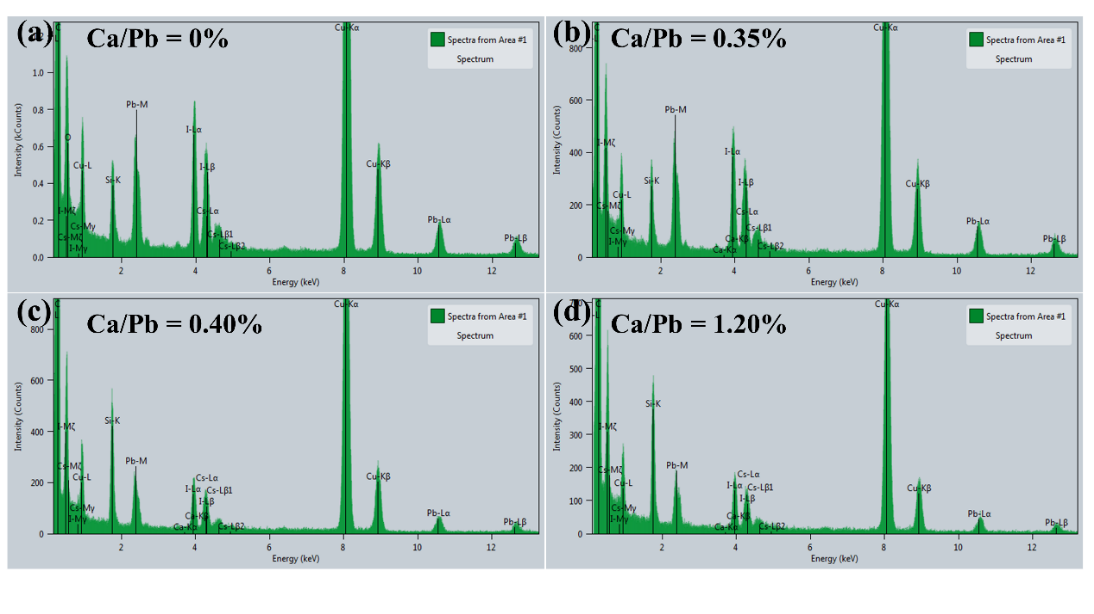


**Figure S3**. EDX spectrum of Ca^2+^-doped CsPbI_3_ NCs. (a) Ca/Pb=0%, (b) Ca/Pb=0.35%, (c) Ca/Pb=0.40%, (d) Ca/Pb=1.20%.


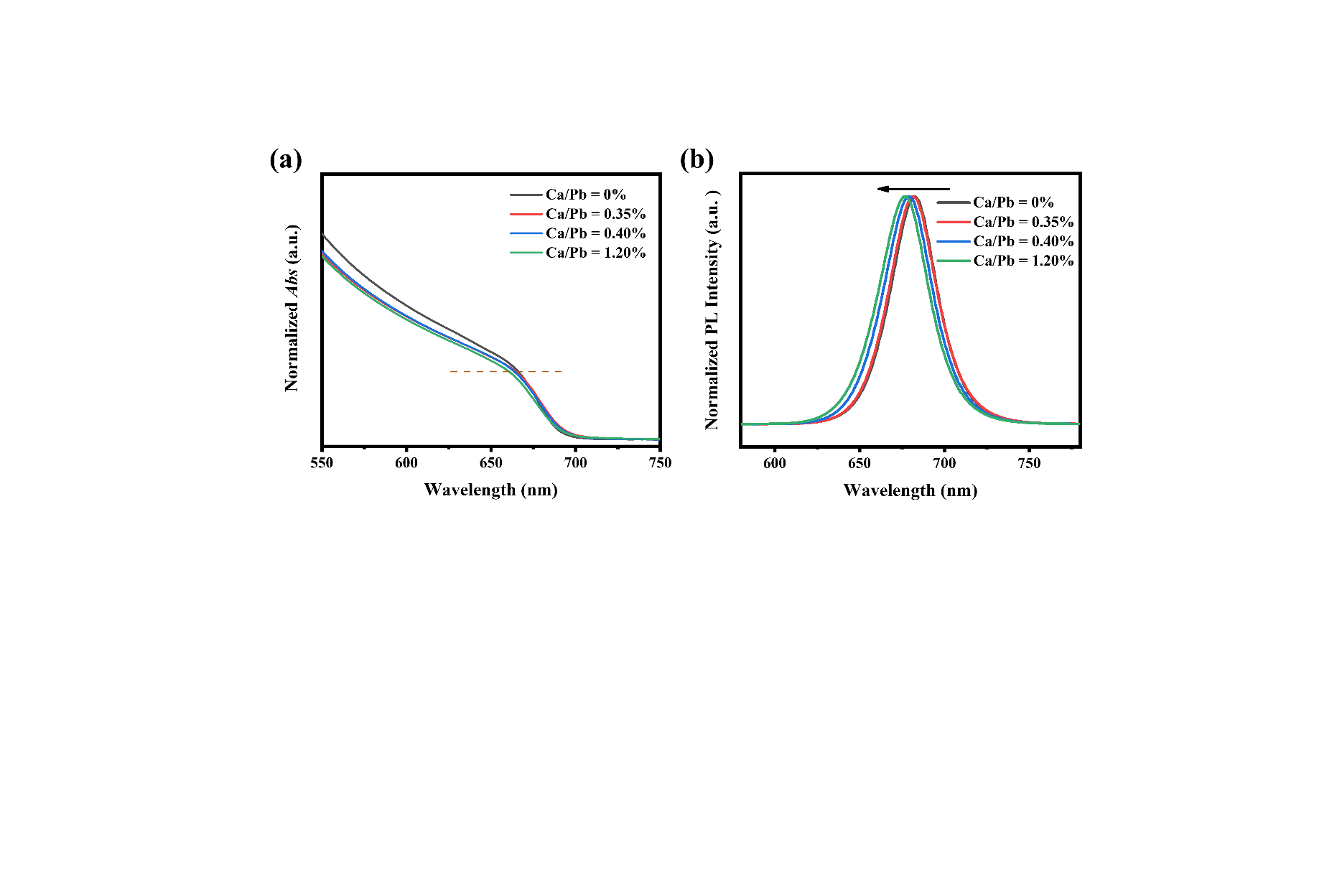


**Figure S4**. (a) Normalized UV-vis absorption (b) Normalized PL spectra of Ca^2+^-doped CsPbI_3_ NCs with different Ca/Pb ratios. (Ca/Pb = 0%, 0.35%, 0.40%, 1.20%)


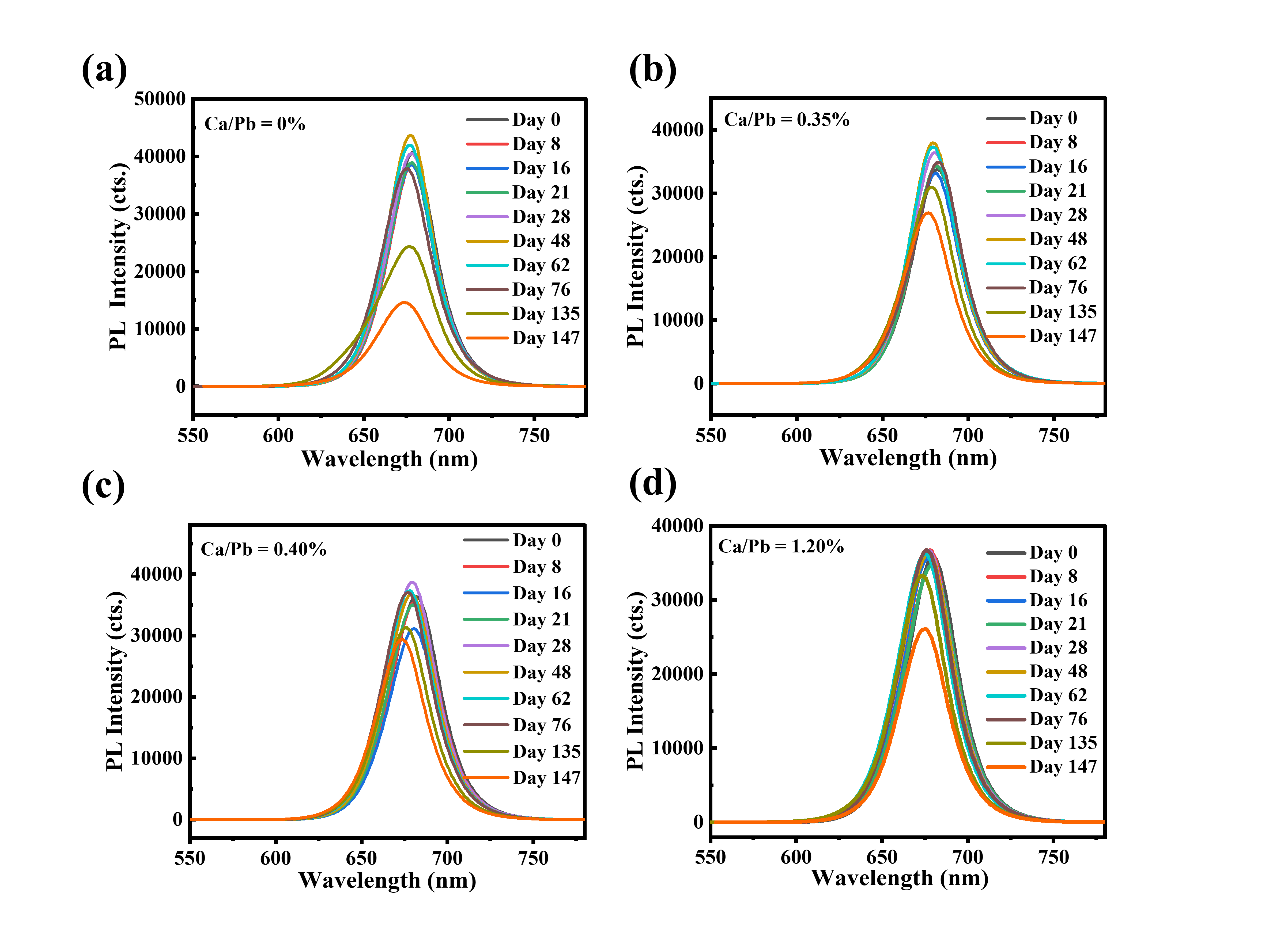


**Figure S5**. The PL evolution of Ca^2+^-doped CsPbI_3_ NCs solutions with increase of storing time. (a) Ca/Pb=0%, (b) Ca/Pb=0.35%, (c) Ca/Pb=0.40%, (d) Ca/Pb=1.20%.


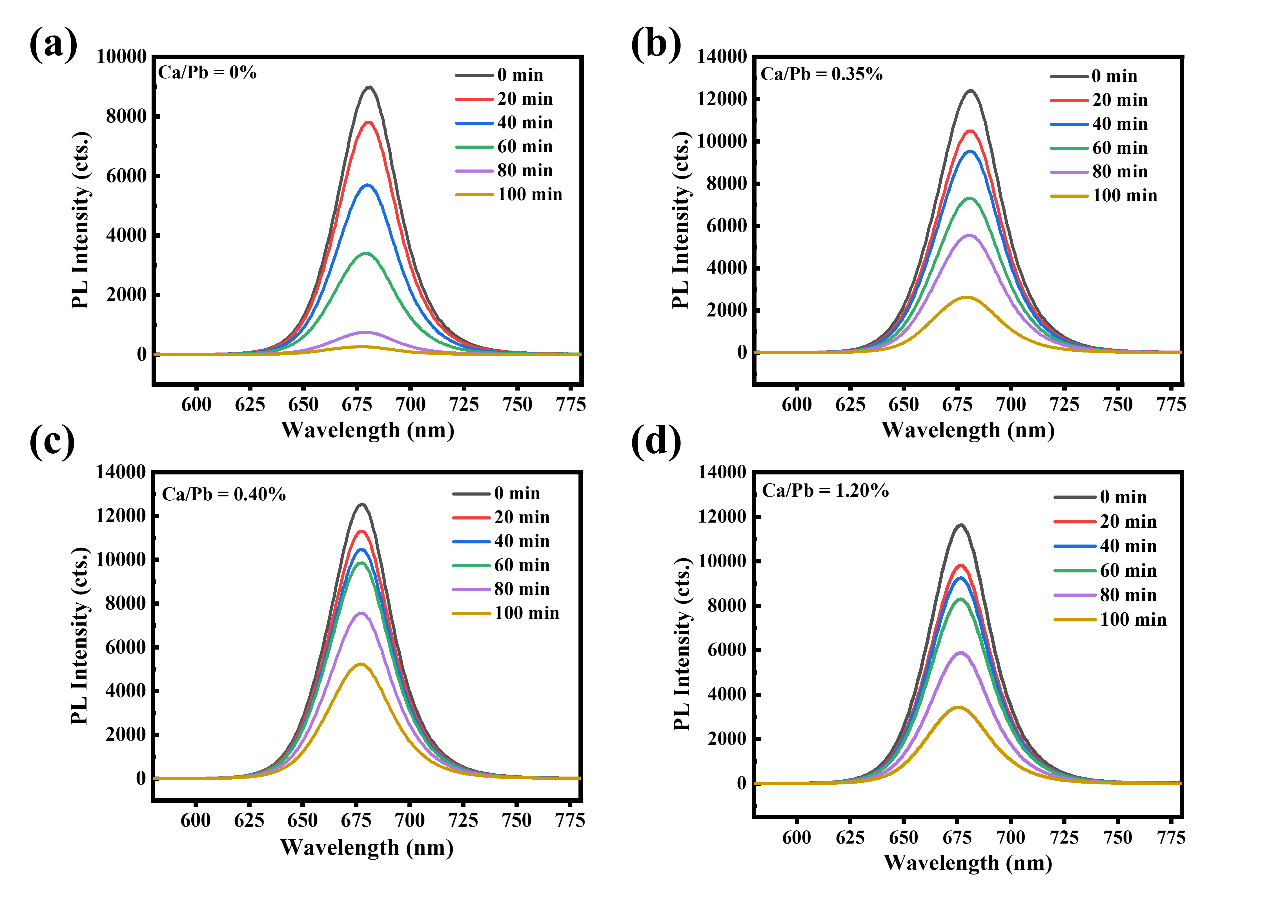


**Figure S6**. The PL areas evolution with increasing UV irradiation time. (a) Ca/Pb = 0%, (b) Ca/Pb = 0.35%, (c) Ca/Pb =0.40%, (d) Ca/Pb = 1.20%.


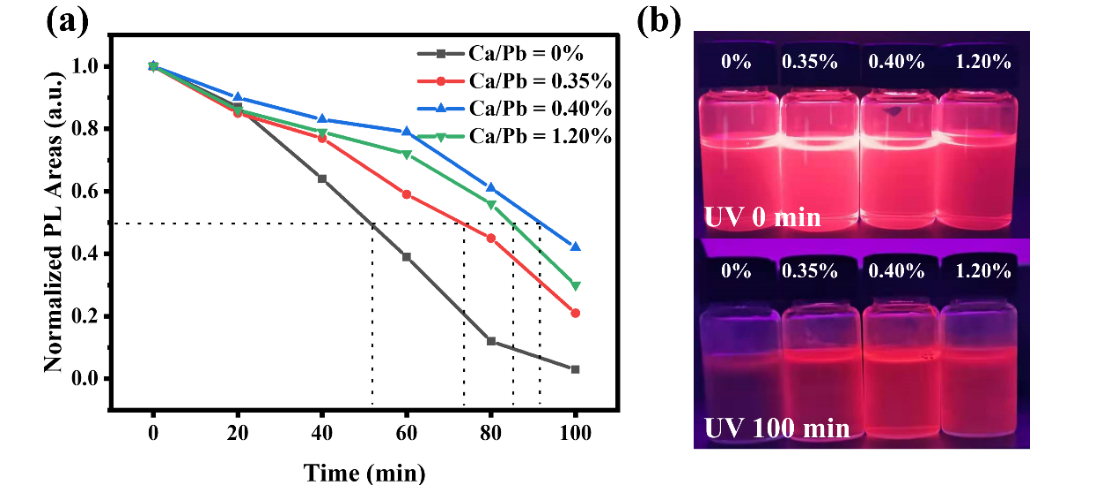


**Figure S7**. The PL evolution of Ca^2+^-doped CsPbI_3_ NCs under 365 nm (8 W) UV irradiation. (a) Ca/Pb = 0%, (b) Ca/Pb = 0.35%, (c) Ca/Pb =0.40%, (d) Ca/Pb = 1.20%.


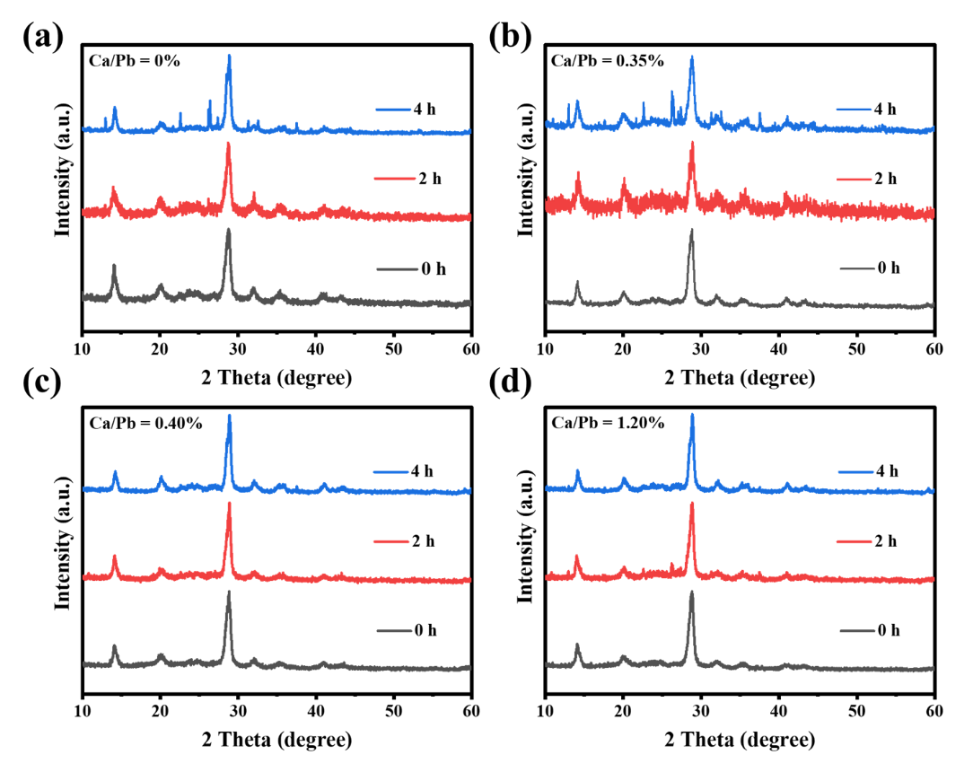


**Figure S8**. XRD patterns evolution for Ca^2+^-doped CsPbI_3_ NCs thin films at 120 ^o^C (humidity 40%-50%). (a) Ca/Pb = 0%, (b) Ca/Pb = 0.35%, (c) Ca/Pb =0.40%, (d) Ca/Pb = 1.20%.


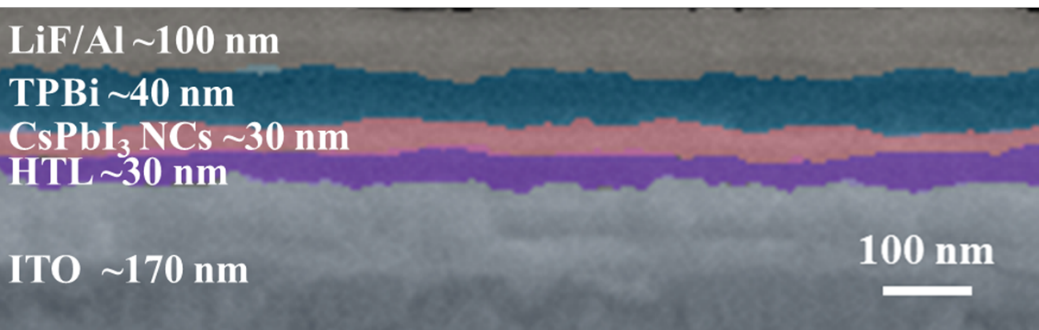


**Figure S9.** The cross-section SEM image of LEDs.


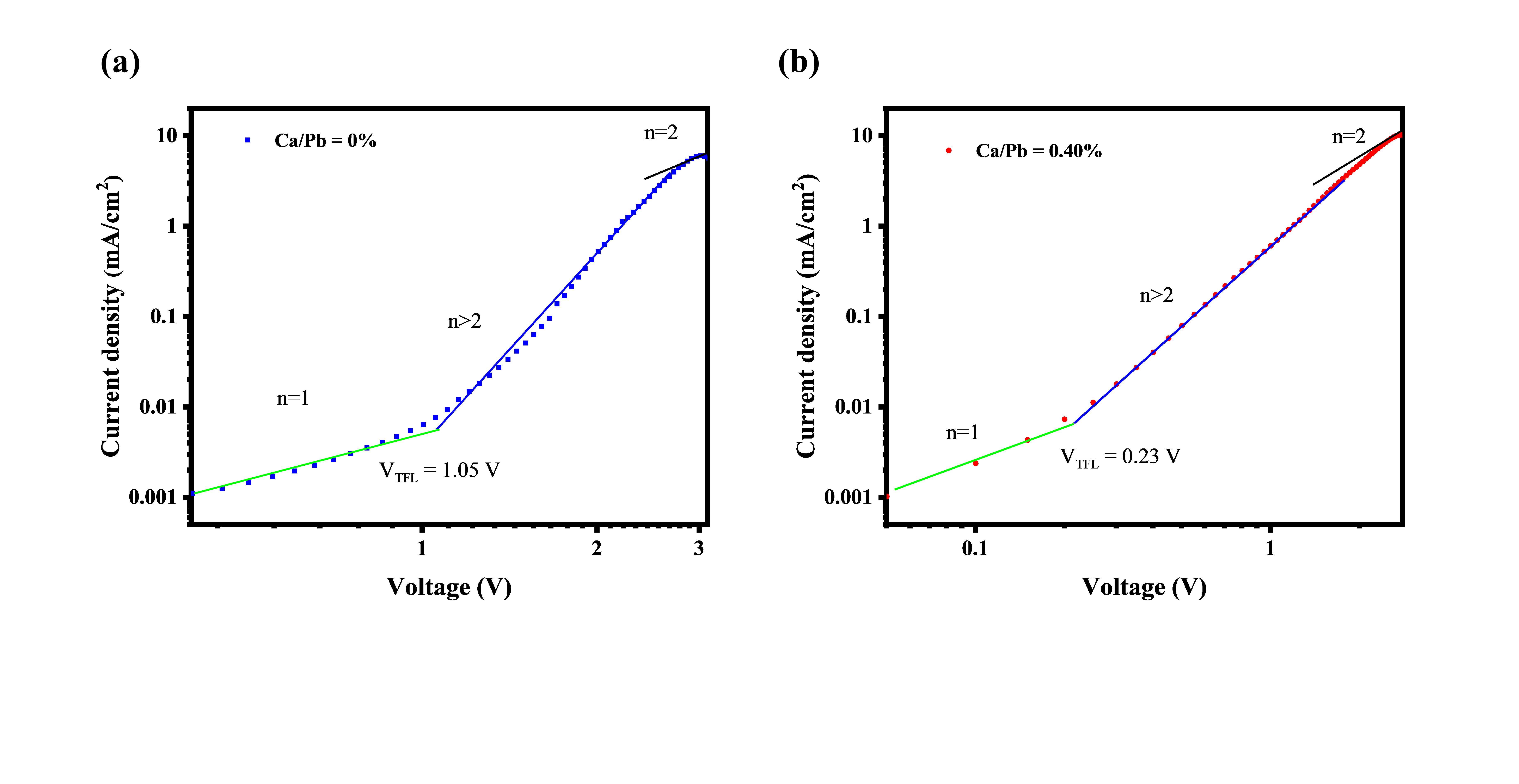


**Figure S10**. *J-V* curves of "hole-only" devices of Ca^2+^-doped CsPbI_3_ NCs. (Ca/Pb =0%, 0.40%)


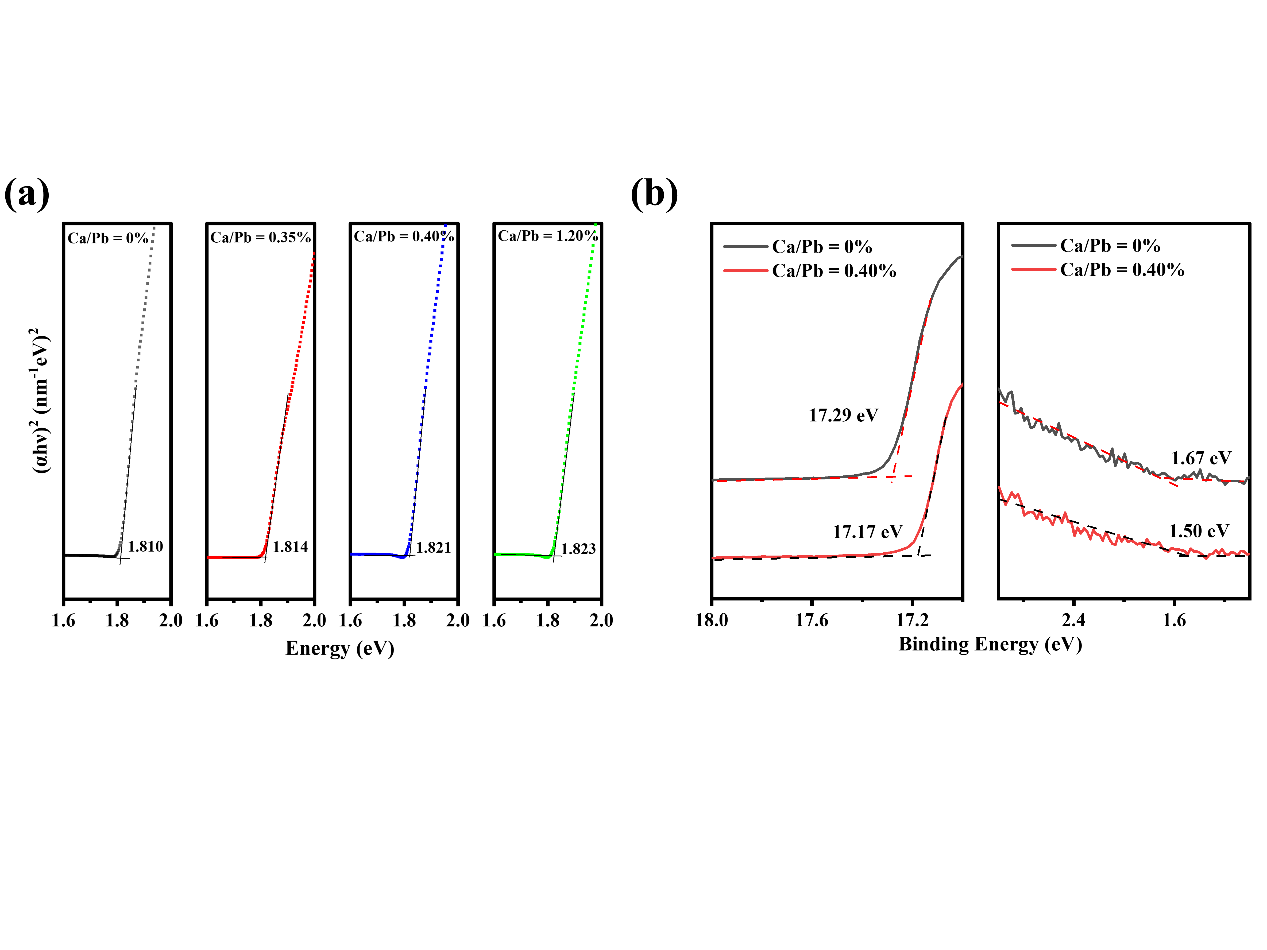


**Figure S11**. (a) E_g_ of Ca^2+^-doped CsPbI_3_ NCs. (b) UPS spectra of Ca^2+^-doped CsPbI_3_ NCs. (Ca/Pb =0%, 0.40%)


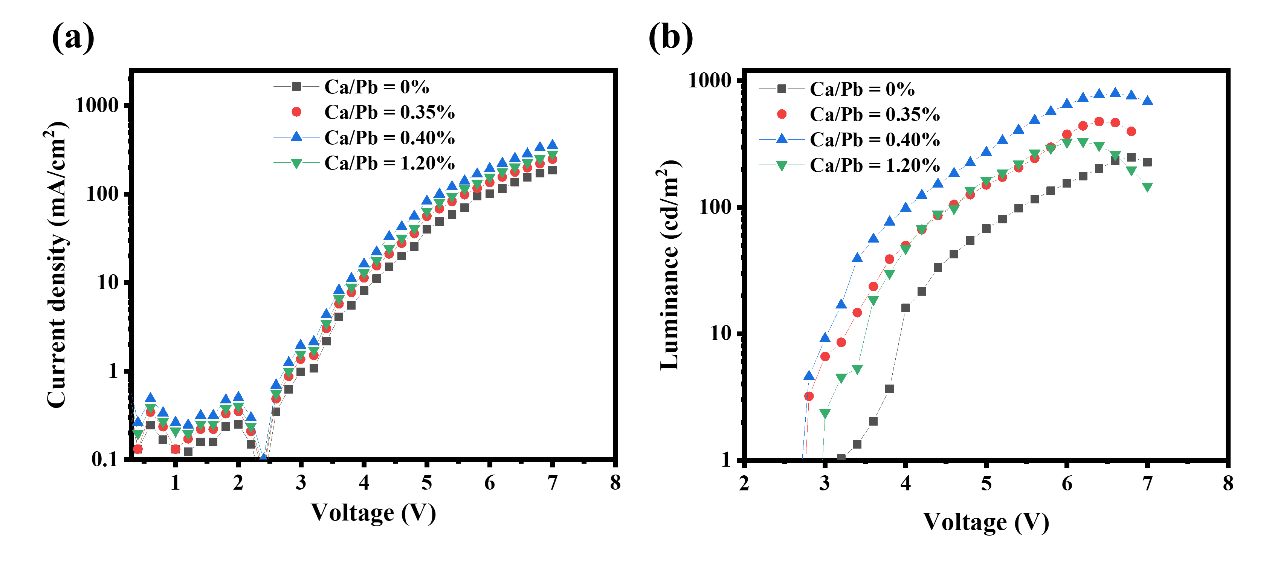


**Figure S12**. (a) current density, (b) luminescence for LEDs.


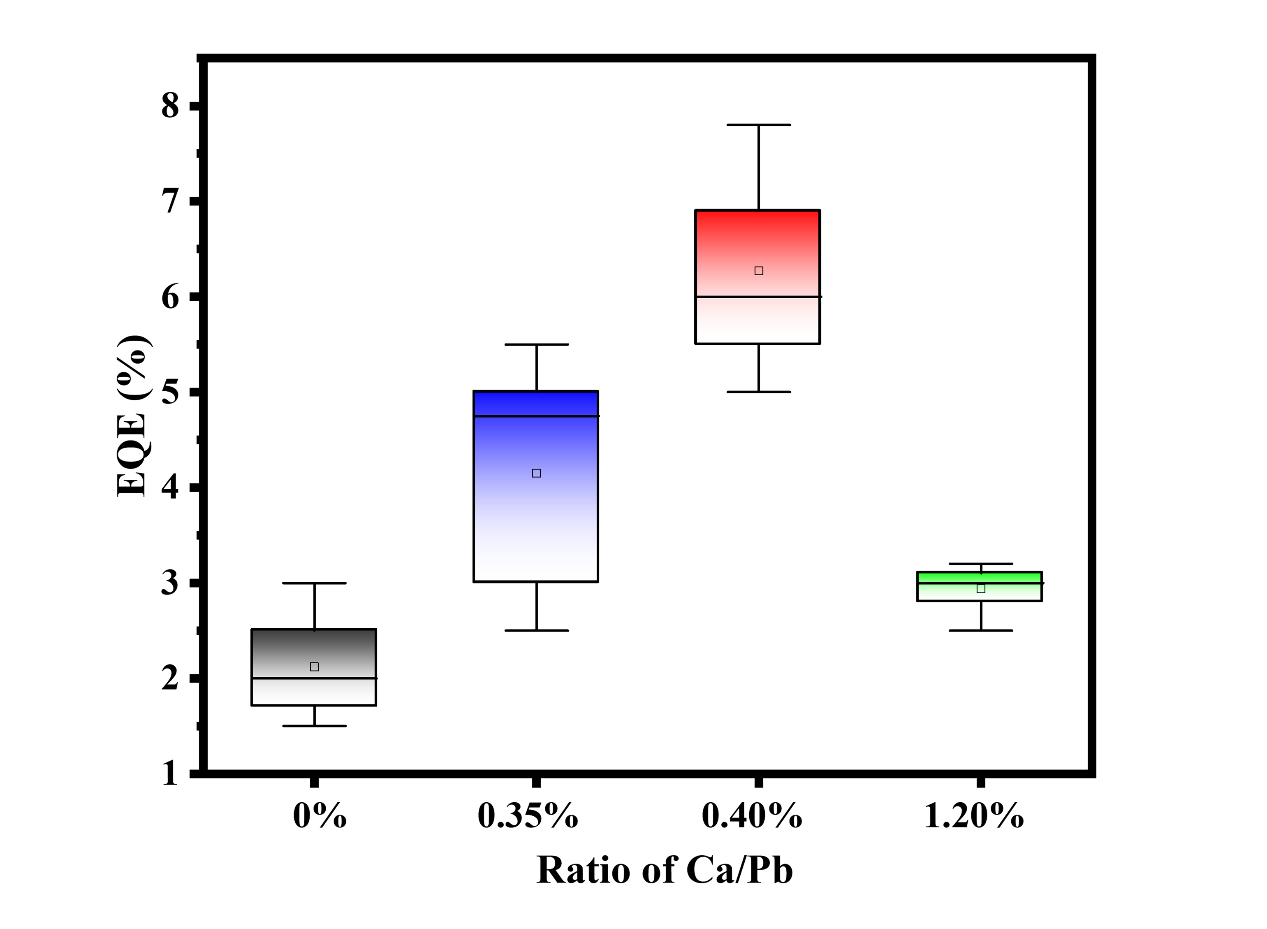


**Figure S13.** Histogram of EQEs for 10 LEDs fabricated by using Ca^2+^-doped CsPbI_3_ NCs as the red-emitting layer.


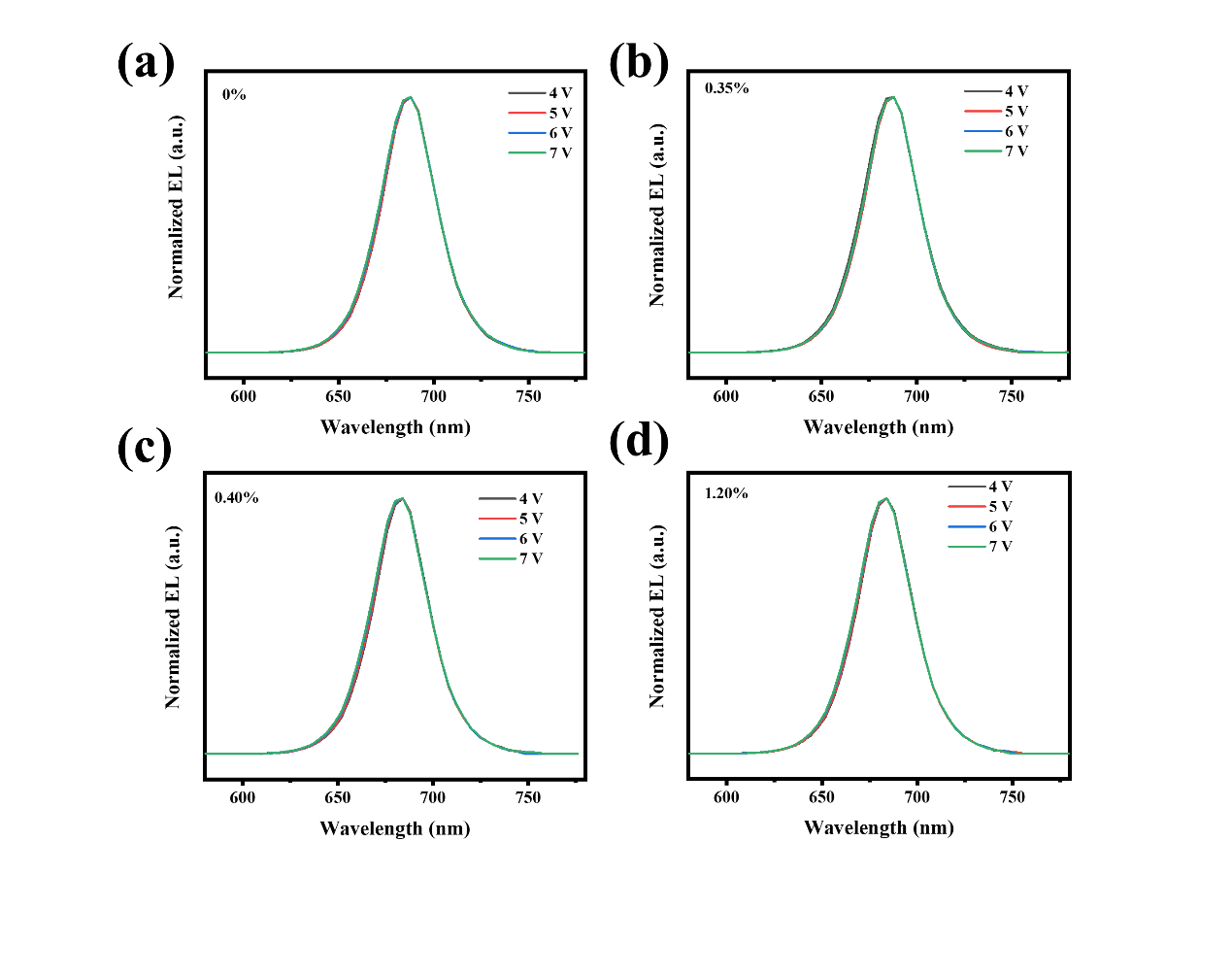


**Figure S14**. EL spectra of LEDs at different voltages.


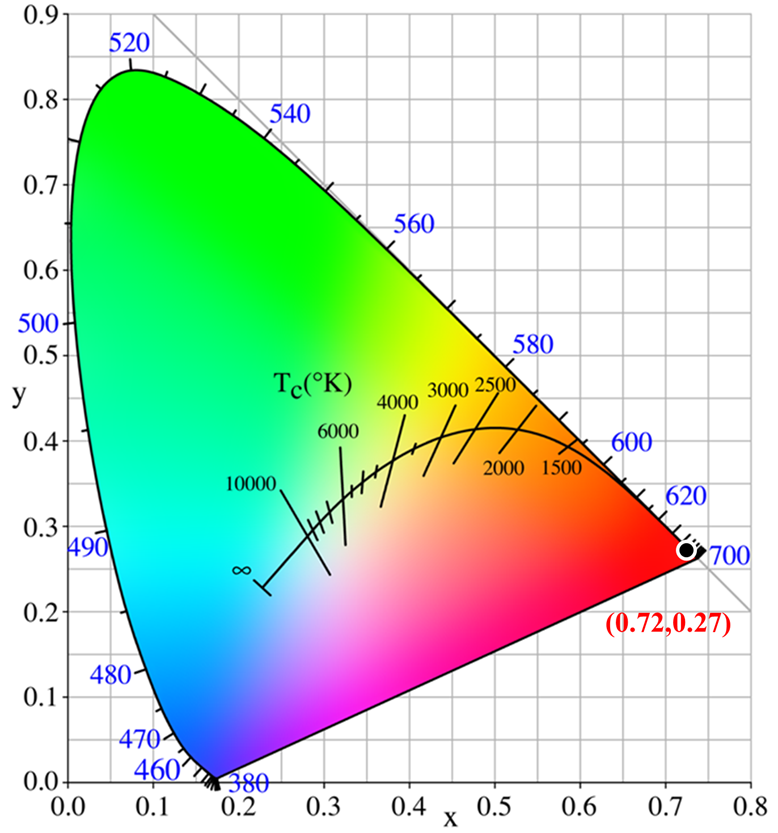


**Figure S15**. The inset shows the corresponding CIE coordinates for the EL spectra.

**Table S1**. The actual ratio of the Ca/Pb by ICP-MS for different Ca/Pb feed ratios

| Ca/Pb feed ratios | Actual ratio of Ca/Pb ratios |
| --- | --- |
| Ca/Pb = 0% | 0% |
| Ca/Pb = 15% | 0.35% |
| Ca/Pb = 25% | 0.40% |
| Ca/Pb = 35% | 1.20% |

**Table S2**. The time-resolved PL decays of samples with Ca^2+^-doped CsPbI_3_ NCs

| Samples | τ_1_(ns) | A_1_ (%) | τ_2_(ns) | A_2_ (%) | τ_avg_(ns) |
| --- | --- | --- | --- | --- | --- |
| Ca/Pb = 0% | 49.07 | 73 | 133.12 | 27 | 71.76 |
| Ca/Pb= 0.35% | 47.75 | 73 | 157.84 | 27 | 77.47 |
| Ca/Pb = 0.40% | 60.83 | 62 | 215.42 | 38 | 119.41 |
| Ca/Pb = 1.20% | 55.77 | 73 | 192.27 | 27 | 92.62 |

**Table S3** Current CsPbI_3_ NCs LED performance with similar structure

| Device structure | V_on_ (V) | L_max_ (cd/m^2^) | EQE(%) | Ref. |
| --- | --- | --- | --- | --- |
| ITO/PEDOT:PSS/Poly-TPD/IDA-CsPbI_3_/TPBi/LiF/Al | 4.5 | 748 | 5.02 | S1 |
| ITO/PEDOT:PSS/Poly-TPD/TMSI-CsPbI_3_/TPBi/LiF/Al | 2.8 | 365 | 1.8 | S2 |
| ITO/PEDOT:PSS/Poly-TPD/DDAB-CsPbI_3_/TPBi/LiF/Al | 4.0 | 468 | 1.25 | S3 |
| ITO/PEDOT:PSS/Poly-TPD/Ni^2+^: CsPbI_3_/TPBi/LiF/Al | 3.3 | 830 | 7.0 | S4 |
| ITO/PEDOT:PSS/Poly-TPD/Sr^2+^: CsPbI_3_/TPBi/LiF/Al | --- | 1250 | 5.92 | S5 |
| ITO/PEDOT:PSS/Poly-TPD/L-PHE-CsPbI_3_/TPBi/LiF/Al | --- | 407 | 10.2 | S6 |
| ITO/PEDOT:PSS/Poly-TPD/Cu^2+^-CsPbI_3_/TPBi/LiF/Al | 1.6 | 1270 | 2.03 | S7 |
| ITO/PEDOT:PSS/Poly-TPD/Sr^2+^:CsPbI_3_/TPBi/LiF/Al | 3.6 | 586 | 17.1 | S8 |
| ITO/PEDOT:PSS/Poly-TPD/PMA- CsPbI_3_/TPBi/LiF/Al | --- | 618 | 17.8 | S9 |
| ITO/PEDOT:PSS/Poly-TPD/PMA- CsPbI_3_/TPBi/LiF/Al | 2.8 | 1000 | 23 | S10 |
| ITO/PEDOT:PSS/Ca^2+^ -CsPbI_3_/TPBi/LiF/Al | 2.8 | 790 | 7.8 | This work |

[S1] J. Pan, Y. Shang, J. Yin, et al., "Bidentate Ligand-Passivated CsPbI_3_ Perovskite Nanocrystals for Stable Near-Unity Photoluminescence Quantum Yield and Efficient Red Light-Emitting Diodes," *Journal of the American Chemical Society*, vol. 140, no. 2, pp. 562-565, 2018.

[S2] Y. Cai, H. Wang, Y. Li, et al., "Trimethylsilyl Iodine-Mediated Synthesis of Highly Bright Red-Emitting CsPbI_3_ Perovskite Quantum Dots with Significantly Improved Stability," *Chemistry of Materials*, vol. 31, no. 3, pp. 881-889, 2019.

[S3] Y. Huang, W. Luan, M. Liu, et al., "DDAB-assisted synthesis of iodine-rich CsPbI_3_ perovskite nanocrystals with improved stability in multiple environments," Journal of Materials Chemistry C, vol. 8, no. 7, pp. 2381-2387, 2020.

[S4] M. Liu, N. Jiang, H. Huang, et al., "Ni^2+^-doped CsPbI_3_ perovskite nanocrystals with near-unity photoluminescence quantum yield and superior structure stability for red light-emitting devices," Chemical Engineering Journal, vol. 413, pp. 127547, 2021.

[S5] J. S. Yao, J. Ge, K. H. Wang, et al., "Few-Nanometer-Sized alpha-CsPbI_3_ Quantum Dots Enabled by Strontium Substitution and Iodide Passivation for Efficient Red-Light Emitting Diodes," Journal of the American Chemical Society, vol. 141, no. 5, pp. 2069-2079, 2019.

[S6] J. Shi, F. Li, Y. Jin, et al., "In Situ Ligand Bonding Management of CsPbI_3_ Perovskite Quantum Dots Enables High-Performance Photovoltaics and Red Light-Emitting Diodes," Angewandte Chemie International Edition, vol. 59, no. 49, pp. 22230-22237, 2020.

[S7] Z. Chen, B. Zhou, J. Yuan, et al., "Cu^2+^-Doped CsPbI_3_ Nanocrystals with Enhanced Stability for Light-Emitting Diodes," The Journal of Physical Chemistry Letters, vol. 12, no. 12, pp. 3038-3045, 2021.

[S8] C. Chen, T. Xuan, W. Bai, et al., "Highly stable CsPbI_3_:Sr^2+^ nanocrystals with near-unity quantum yield enabling perovskite light-emitting diodes with an external quantum efficiency of 17.1%," Nano Energy, vol. 85, pp. 106033, 2021.

[S9] H. Li, H. Lin, D. Ouyang, et al., "Efficient and Stable Red Perovskite Light-Emitting Diodes with Operational Stability >300 h," Advanced Materials, vol. 33, no. 15, pp. 2008820, 2021.

[S10] Y. K. Wang, F. Yuan, Y. Dong, et al., "All-Inorganic Quantum-Dot LEDs Based on a Phase-Stabilized alpha-CsPbI_3_ Perovskite," Angewandte Chemie International Edition, vol. 60, no. 29, pp. 16164-16170, 2021.
